# Universal and culture-tuned neural codes for vocal emotion: an fMRI MVPA study using Japanese and Canadian voices

**DOI:** 10.1093/oons/kvag001

**Published:** 2026-03-17

**Authors:** Michihiko Koeda, Bastien Cagna, Tomoko Hama, Yoshiro Okubo, Amane Tateno, Pascal Belin

**Affiliations:** Department of Neuropsychiatry, Nippon Medical School Tama Nagayama Hospital, Tokyo, Japan; Department of Neuropsychiatry, Graduate School of Medicine, Nippon Medical School, Tokyo, Japan; Timone Neuroscience Institute, CNRS and Aix-Marseille University, Marseille, France; Department of Medical Technology, Ehime Prefectural University of Health Sciences, Ehime, Japan; Department of Neuropsychiatry, Graduate School of Medicine, Nippon Medical School, Tokyo, Japan; Department of Neuropsychiatry, Graduate School of Medicine, Nippon Medical School, Tokyo, Japan; Timone Neuroscience Institute, CNRS and Aix-Marseille University, Marseille, France

**Keywords:** emotion recognition, Fmri, Mvpa, auditory cortex, cross-cultural

## Abstract

Understanding how the human brain decodes nonverbal emotional voices is essential for elucidating the neural basis of social cognition and for advancing research on affective dysfunction in psychiatric disorders. Using functional MRI and multivariate pattern analysis (MVPA), we investigated both universal and culture-tuned neural mechanisms underlying emotional voice perception. Forty-one healthy adults (22 Asian, 19 Western participants) listened to five emotions—angry, sad, neutral, happy, and pleased—expressed by Japanese (Tokyo Affective Voices, TAV) and Canadian (Montreal Affective Voices, MAV) actors. Whole-brain MVPA revealed robust decoding in bilateral superior temporal gyri, inferior and middle frontal gyri, anterior cingulate cortex, and insula. Sadness showed the most stable representation, whereas pleasure engaged medial prefrontal and cingulate regions, reflecting internally oriented processing of positive affect. Modest cultural effects emerged, with higher decoding for neutral and happy TAV stimuli. These findings suggest a framework for objectively characterizing emotional voice processing across cultural contexts. Such an approach may provide a basis for future translational research aimed at informing more objective approaches to the study of social and affective functioning, while further validation will be required.

## Introduction

Recent advances in neuroimaging now allow the decoding of social- and emotion-related representations from distributed brain activity (e.g. MVPA), offering promising approaches for the objective characterization of affective processing in psychiatric research. However, whether—and how—these neural codes vary across cultures and for nonverbal auditory emotions remains insufficiently understood.

Understanding how humans recognize and express emotion is fundamental for social communication and mental health (Cowen et al. 2021; Elfenbein and Ambady 2002; Schirmer and Kotz 2006). In psychiatric practice, clinicians often encounter patients whose emotional expressions are misunderstood or misinterpreted by others (Cotter et al. 2018; Edwards et al. 2002). This difficulty in mutual understanding highlights the challenge of decoding emotions beyond verbal content, relying on nonverbal communication cues (Elfenbein and Ambady 2002; Juslin and Laukka 2003). Vocal tone conveys affective meaning even without words, revealing emotional states that facial or linguistic signals may not capture (Schirmer and Kotz 2006; Belin et al. 2008). Research on vocal emotion processing shows that specific brain regions, including the superior temporal gyrus (STG), are critical for decoding affective voices (Schirmer and Kotz 2006; Fecteau et al. 2004). However, how these neural mechanisms differ across emotions and cultures remains insufficiently understood (Koeda et al. 2013; Sauter et al. 2010).

Previous studies using the Montreal Affective Voices (MAV) demonstrated reliable recognition of basic emotions, such as sadness and anger, across Western listeners (Belin et al. 2008; Pell et al. 2009). The Tokyo Affective Voices (TAV) were recently developed to provide culturally relevant emotional vocalizations by Japanese actors, enabling East–West comparison (Koeda et al. 2013; Koeda et al. 2025). Behavioral studies suggest that negative emotions like sadness are recognized universally, while positive emotions such as pleasure vary across cultural contexts (Sauter et al. 2010; Jiang et al. 2023). Yet, the neural representation of these universal and culture-specific aspects of emotional voice processing has not been clearly delineated (Schirmer and Kotz 2006; Fecteau et al. 2004).

Recent advances in neuroimaging have enabled fine-grained decoding of emotional perception from distributed brain activity (Schirmer and Kotz 2006; Neumann et al. 2022). Understanding the neural decoding of emotional voices has both theoretical and clinical relevance (Cowen et al. 2021; Fecteau et al. 2004). Impaired emotion recognition is a hallmark of several psychiatric disorders, including depression, schizophrenia, autism spectrum disorder, and dementia (Cotter et al. 2018; Edwards et al. 2002). In clinical practice, clarifying how emotional processing relates to the brain’s capacity for empathy and communication is crucial for elucidating the neural basis of social cognition (Schirmer and Kotz 2006; Pell et al. 2009). Therefore, identifying how the healthy brain decodes emotional voices could provide a foundation for future efforts aimed at characterizing objective markers related to social functioning (Belin et al. 2008; Koeda et al. 2013).

Functional MRI combined with multivariate pattern analysis (MVPA) allows for the decoding of distributed neural patterns underlying emotion perception (Haynes and Rees 2006; Norman et al. 2006). By comparing TAV and MAV stimuli across Asian and Western participants, this study examined both shared and culturally tuned neural representations of emotional voice processing. We hypothesized that sadness and happiness would show higher decoding accuracy than anger or pleasure, reflecting more stable neural representations. We also predicted subtle cultural effects, with TAV recognized more accurately by Asian participants and MAV by Western participants. Ultimately, this study aims to reveal how universal and culture-tuned neural mechanisms jointly shape emotional voice perception and inform psychiatric research. Accordingly, we leveraged culturally validated vocal sets (TAV/MAV) and whole-brain MVPA to test these predictions while explicitly modeling cultural influences on nonverbal voice processing.

## Methods

### Participants

Twenty-two East Asian right-handed participants (10 males and 12 females; mean age = 25.2 years, SD = 3.6) and 19 European Western right-handed participants (7 males and 12 females; mean age = 26.1 years, SD = 4.9) took part in the present study.

The Asian participants included 10 Chinese 9 Japanese 1 Malaysian 1 Thai, and 1 Taiwanese individual. The Western participants included 9 British 4 German 1 Spanish 1 Polish 1 Russian 1 Estonian 1 Italian, and 1 Romanian individual.

All participants were free from any history of psychiatric disorder, significant physical illness, head injury, neurological disorder, or alcohol or drug dependence.

The mean years of education were 16.0 ± 0.8 for the Asian group and 15.6 ± 0.6 for the Western group, with no significant difference between them (Welch’s two-sample t-test, t(35.13) = 1.70, p = 0.097, two-tailed). All participants were right-handed. The mean score on the Edinburgh Handedness Inventory (EHI) (Oldfield 1971) was 91.1 ± 9.3 for the Asian group and 93.1 ± 6.7 for the Western group, with no significant difference between them (Welch’s two-sample t-test, t(34.39) = −0.79, p = 0.436, two-tailed). The mean duration of residence in the United Kingdom among Asian participants was 5.0 ± 2.9 months.

After a complete explanation of the study, written informed consent was obtained from all participants. The study was approved by the Ethics Committee of the relevant institutional ethics committee. Participants received £12 for their time (£6 per hour).

### Experiment design

**Figure 1:**
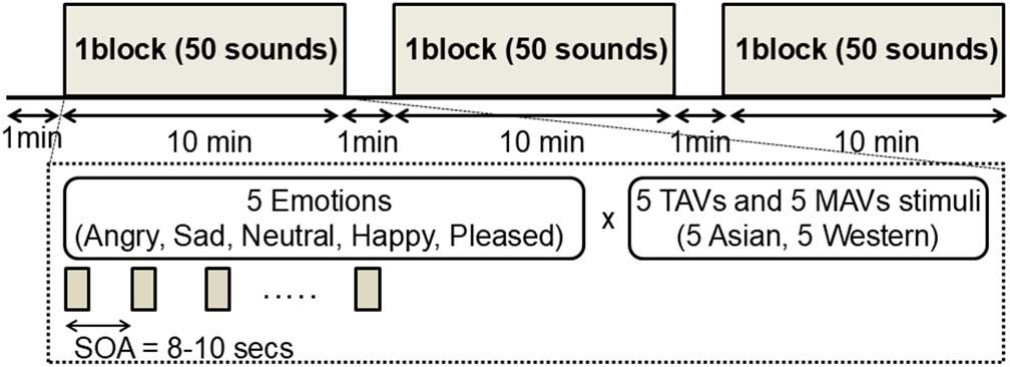
Experimental protocol of the fMRI study. Each session consisted of three sets of a resting period (1 min) and a task block (10 min) presented in a pseudo-randomized order. During each block, participants listened to 50 emotional vocalizations (angry, sad, neutral, happy, pleased). MAV = Montreal affective voices; TAV = Tokyo affective voices; SOA = stimulus-onset asynchrony

The total duration of the fMRI experiment was 33 minutes. Each session consisted of three sets of a resting period (1 min) and a task block (10 min), presented in a pseudo-randomized order (Fig. 1). During each task block, participants listened to 50 nonverbal emotional vocalizations, comprising five emotions—angry, sad, neutral, happy, and pleased—expressed by ten actors (five Asian and five Western participants).

The stimuli were drawn from the Montreal Affective Voices (MAV; (Belin et al. 2008)) and the Tokyo Affective Voices (TAV; see Stimuli section for details (Koeda et al. 2025)).

Before scanning, each participant received a reference sheet describing the definitions of six basic emotions (angry, disgust, fear, happy, sad, and pleasure), based on a previous cross-cultural study (Sauter et al. 2010).

The definitions were summarized as follows: happy – *joyful, glad, delighted*; pleasure – amusement, *diversion, or worldly enjoyment*; angry – *furious, very annoyed, irritable*; disgust – *strong distaste or aversion*; fear – *frightened, scared*; sad – *unhappy or feeling down* (see Supplement Table 1).

Only participants who achieved a perfect score (8/8 items) on this pre-scanning emotion-definition check proceeded to the fMRI task (see Supplement Table 2). During scanning, participants listened to the emotional vocalizations and judged the emotional valence of each sound using a button press (positive = 3, neutral = 2, negative = 1). Stimuli were presented in a pseudo-randomized order, with stimulus-onset asynchrony (SOA) jittered between 8 and 10 s. A total of 50 emotional stimuli were presented per block.

### Stimuli

The stimuli were selected from the Tokyo Affective Voices (TAV; (Koeda et al. 2025)) and the Montreal Affective Voices (MAV; (Belin et al. 2008)).

Each set consisted of nonverbal emotional vocalizations produced by ten professional actors (five East Asian and five European), expressing five target emotions—angry, sad, neutral, happy, and pleased.

All recordings were made in a sound-attenuated studio using a digital recorder (44.1 kHz sampling rate, 16-bit resolution).

Each vocalization was edited to remove onset/offset noise and normalized to the same root-mean-square (RMS) amplitude using Praat (Boersma and Weenink 2024).

Stimulus duration ranged from 1.0 to 1.5 s (mean = 1.2 s).

**Figure 2:**
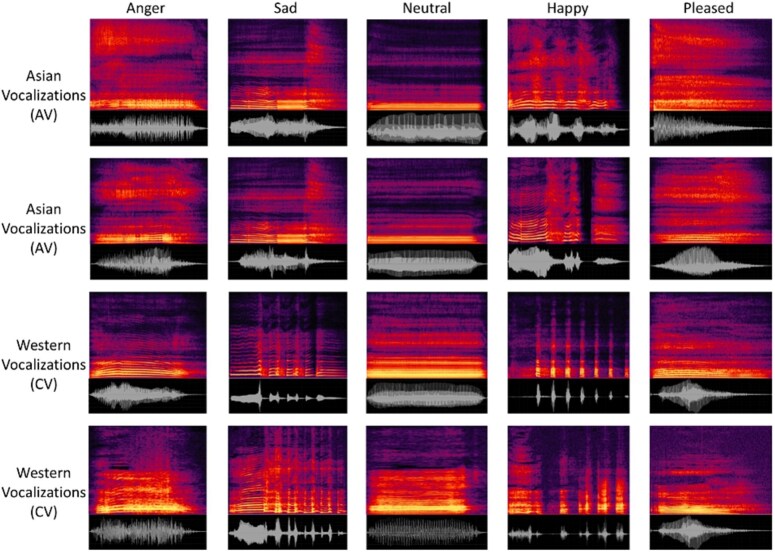
Example spectrograms and waveforms of the emotional stimuli. Time (horizontal axis) and frequency (vertical axis, 0–16 kHz) are shown for representative vocalizations from the MAV and TAV sets. Amplitude (vertical axis in waveform) is shown in samples (−10 000 to 12 500 smpl)

The final stimulus set was matched across TAV and MAV in total number, duration, and intensity. Representative spectrograms and waveforms of the emotional vocalizations from the TAV and MAV sets are shown in Fig. 2.

### Pre-scanning validation

Before the fMRI session, participants completed a short pre-task to confirm their conceptual understanding of the emotion categories.

Each participant was asked to match words to definitions of seven emotions—angry, disgust, fear, happy, sad, pleasure, and neutral—based on Sauter et al. (Sauter et al. 2010) (Sauter et al. 2010).

The definitions were summarized as follows: happy – joyful, glad, delighted; pleasure – *amusement, diversion, enjoyment*; angry – *furious, annoyed*; disgust – *loathing*; fear – *frightened*; sad – *unhappy*; neutral – *non-emotional feeling*.

Performance accuracy exceeded 95%, confirming that all participants possessed an adequate conceptual understanding of the emotion categories before the fMRI experiment.

### MRI data acquisition

MRI data were acquired using a 3-Tesla Siemens Magnetom Tim Trio scanner (Siemens Medical Solutions, Erlangen, Germany) equipped with a 32-channel head coil at the University of Glasgow.

Functional images were obtained using a T2*-weighted gradient-echo echo-planar imaging (EPI) sequence sensitive to blood oxygen level–dependent (BOLD) contrast (repetition time [TR] = 2000 ms; echo time [TE] = 30 ms; flip angle = 90°; field of view [FOV] = 192 × 192 mm; matrix = 64 × 64; slice thickness = 3 mm; interslice gap = 0.3 mm; 36 axial slices; voxel size = 3 × 3 × 3 mm^3^).

Each functional run consisted of 300 volumes, with three runs per participant (total = 900 volumes).

A high-resolution T1-weighted structural image was also acquired for anatomical reference (magnetization-prepared rapid gradient-echo [MPRAGE] sequence: TR = 2530 ms; TE = 3.03 ms; flip angle = 7°; FOV = 256 × 256 mm; matrix = 256 × 256; 176 sagittal slices; voxel size = 1 × 1 × 1 mm^3^).

During scanning, participants lay supine in the scanner, and head motion was minimized using foam padding.

Auditory stimuli were presented through MRI-compatible headphones (NordicNeuroLab, Bergen, Norway), and responses were recorded using an MRI-compatible button box.

### Image preprocessing

Image preprocessing and statistical analyses were performed using SPM12 (Wellcome Centre for Human Neuroimaging, London, UK) implemented in MATLAB R2022b (MathWorks, Natick, MA, USA) (Penny et al. 2011).

Functional images were first corrected for differences in slice acquisition timing and realigned to the first volume of each run to correct for head motion.

The mean functional image was coregistered to the corresponding structural T1-weighted image, which was then segmented into gray matter, white matter, and cerebrospinal fluid using the unified segmentation approach.

Individual flow fields generated from the segmentation were used for DARTEL (Ashburner 2007) to create a study-specific template and to normalize all functional images into the MNI space with 3 × 3 × 3 mm^3^ isotropic voxels.

The normalized functional images were spatially smoothed with an 8-mm full-width-at-half-maximum (FWHM) Gaussian kernel to improve the signal-to-noise ratio.

Temporal high-pass filtering (cutoff = 128 s) was applied to remove low-frequency drifts.

### Multivariate pattern analysis (MVPA)

MVPA was conducted to examine distributed neural representations of emotional vocalizations using a whole-brain searchlight approach (Kriegeskorte et al. 2006) implemented in MATLAB R2022b (MathWorks, Natick, MA, USA) and SPM12 (Wellcome Centre for Human Neuroimaging, London, UK).

For each participant, voxelwise activation patterns from preprocessed BOLD images were analyzed within spherical searchlights (radius = 8 mm) using linear support vector machines (SVMs; (Cortes and Vapnik 1995)).

Classification was performed using in-house MATLAB scripts based on SPM12 functions together with the LIBSVM library (Chang and Lin 2011), implementing a linear SVM with default parameters (cost parameter C = 1).

Multi-class classification across the five emotion categories (angry, sad, neutral, happy, and pleased) was implemented using a one-versus-one scheme.

Decoding accuracy was computed using a leave-one-run-out cross-validation scheme across the three functional runs.

For each voxel, decoding accuracy was expressed as raw classification accuracy (%), with the theoretical chance level defined as 20% for five-class classification.

Voxelwise decoding accuracy maps were normalized to MNI space using individual DARTEL flow fields generated during preprocessing and spatially smoothed with an 8-mm full-width-at-half-maximum (FWHM) Gaussian kernel.

These normalized and smoothed decoding accuracy maps were entered into a second-level random-effects analysis using a 2 (Group: Asian, Western; between-subjects) × 2 (Stimulus: TAV, MAV; within-subjects) × 5 (Emotion: angry, sad, neutral, happy, pleased; within-subjects) full factorial design, yielding 20 condition-specific maps per participant.

Voxelwise one-sample t-tests were conducted at the group level to evaluate whether decoding accuracy maps differed significantly from the theoretical chance level (20%) for five-class classification. In addition, full factorial analyses of variance (ANOVAs) were performed to examine the main effects of Group, Stimulus, and Emotion, as well as their interactions, using the standard factorial design module in SPM12. All statistical maps were thresholded at p < 0.05, family-wise error (FWE) corrected at the voxel level, with a cluster-extent threshold of 20 contiguous voxels.

This procedure precisely corresponds to the implementation documented in the publicly archived script (commit_run_fix.m) available via the OSF repository.

The script includes the exact factorial model specifications (2 × 2 × 5 design), image normalization via DARTEL flow fields, spatial smoothing (8 mm FWHM), and random-effects full factorial modeling at the group level.

Results and tables refer to mean accuracy within significant clusters (or peak voxels), and are not themselves used as the statistical threshold.

## Results

### Behavioral results in fMRI experiments

**Figure 3:**
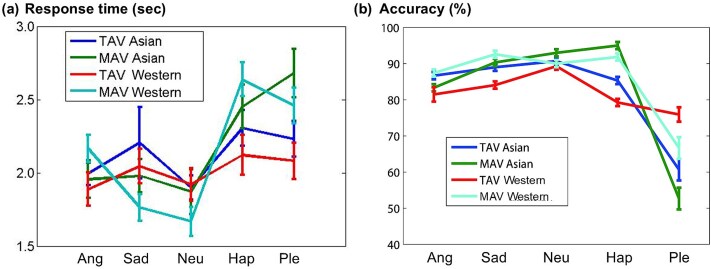
Behavioral results obtained during fMRI experiments. (a) Response time (s) and (b) accuracy (%) for each emotion (ang = angry, sad = sad, neu = neutral, hap = happy, Ple = pleased). Error bars indicate standard errors of the mean. Main effects and interactions were assessed by a three-way mixed ANOVA (group × stimulus × emotion)

Figure 3 summarizes the behavioral results obtained during the fMRI experiments.

Both response time (RT) and accuracy were analyzed using a mixed 2 (Group: Asian, Western; between-subjects) × 2 (Stimulus: TAV, MAV; within-subjects) × 5 (Emotion: angry, sad, neutral, happy, pleased; within-subjects) ANOVA.

In addition, between-group differences in each emotion were assessed using Mann–Whitney U tests, revealing no significant differences between Asian and Western participants in either RT or accuracy (*p* > 0.05).

A significant main effect of Emotion was observed for both RT (*F*(4,144) = 46.4, *p* < 0.001) and accuracy (*F*(1.9,67.5) = 31.1, *p* < 0.001) (see Fig. 3 and Table 1).

**Table 1:** Results of the three-way mixed ANOVA on response time and accuracy during the fMRI experiment

	Response_Time			Accuracy	
	F	p		F	p
Main effect of Emotion	F(4,144) = 46.4	<0.001		F(1.9,67.5) = 31.1	<0.001
Main effect of Group (Asian-Western)	F(1,36) = 0.06	n.s.		F(1,36) = 0.21	n.s.
Main effect of TAVMAV	F(1,36) = 8.59	0.006		F(1,36) = 2.03	n.s.
Interaction effect of Emotion × Group	F(1,36) = 2.94	0.023		F(1.9,67.5) = 2.78	0.03
Interaction effect of Emotion × TAVMAV	F(4,144) = 3.09	0.018		F(3.1111.0) = 7.48	<0.001
Interaction effect of Group ×TAVMAV	F(1,36) = 2.74	n.s.		F(1,36) = 1.32	n.s.
Interaction effect of Emotion × Group × TAVMAV	F(3,34) = 0.28	n.s.		F(3.1111.0) = 0.87	n.s.
	Factor	Response_Time		Accuracy	
		F	p	F	p
Main effect	Emotion	F(4,144) = 46.4	<0.001	F(1.9,67.5) = 31.1	<0.001
	Asian-Western	F(1,36) = 0.06	n.s.	F(1,36) = 0.21	n.s.
	TAV MAV	F(1,36) = 8.59	0.006	F(1,36) = 2.03	n.s.
Interaction effect	Emotion × Asian-Western	F(1,36) = 2.94	0.023	F(1.9,67.5) = 2.78	0.03
	Emotion × TAV MAV	F(4,144) = 3.09	0.018	F(3.1111.0) = 7.48	<0.001
	Asian-Western × TAV MAV	F(1,36) = 2.74	n.s.	F(1,36) = 1.32	n.s.
	Emotion × Asian-Western × TAV MAV	F(3,34) = 0.28	n.s.	F(3.1111.0) = 0.87	n.s.

Factors: Group (Asian/Western) × Stimulus (TAV/MAV) × Emotion (angry, sad, neutral, happy, pleased).

Significant main effects and interactions are indicated (*P* < 0.05).

Post hoc Fisher’s least significant difference (LSD) tests showed that RTs were significantly longer for Sad and Pleased than for Neutral and Happy, whereas accuracy was significantly lower for Pleased compared with the other emotions (*p* < 0.05).

A significant main effect of **Stimulus Type** (TAV vs. MAV) was also found for RT (*F*(1,36) = 8.59, *p* < 0.05), indicating slower responses to TAV than to MAV stimuli.

The main effect of **Group** was not significant for either RT or accuracy (*p* > 0.05).

Significant **Emotion × Group** and **Emotion × Stimulus** interactions were found, suggesting cultural modulation of behavioral responses.

For the **Emotion × Group** interaction, RTs differed between groups (*F*(1,36) = 2.94, *p* < 0.05): Asians responded more slowly than Western participants to *Angry* and *Pleased* voices, but faster to *Sad*, *Neutral*, and *Happy* voices.

Accuracy also exhibited a significant interaction (*F*(1.9,67.5) = 2.78, *p* < 0.05), with Asians being more accurate for *Pleased* and less accurate for *Happy* voices.

For the **Emotion × Stimulus** interaction, RTs were greater for *Neutral* and *Happy* voices in TAV than in MAV, and smaller for *Angry*, *Sad*, and *Pleased* voices (*F*(4,144) = 3.09, *p* < 0.05).

Accuracy showed a similar pattern (*F*(3.1111) = 7.48, *p* < 0.001): *Pleased* voices were recognized more accurately in TAV, whereas *Sad* and *Happy* voices were recognized more accurately in MAV.

No significant three-way interaction (**Emotion × Group × Stimulus**) was observed (*p* > 0.05).

### Neural decoding by MVPA (whole-brain)

Whole-brain multivariate pattern analysis (MVPA) was performed using searchlight decoding of emotion categories across all voxels (see Methods, Section 2.7).

**Figure 4:**
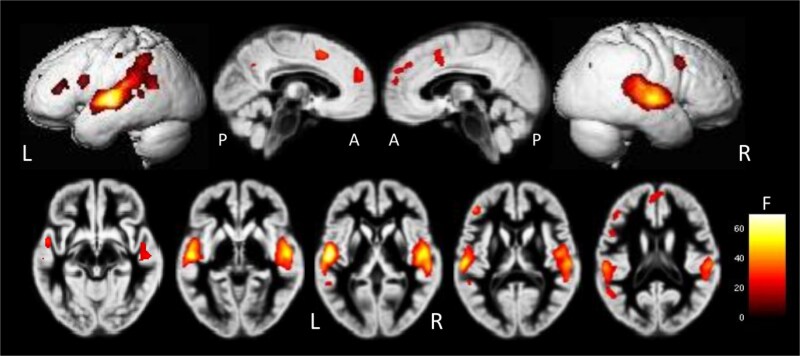
Whole-brain decoding accuracy maps obtained by multivariate pattern analysis (MVPA). Results of the 2 (group: Asian, western) × 2 (stimulus: TAV, MAV) × 5 (emotion) full-factorial analysis. Regions showing significant decoding effects included the bilateral superior temporal gyrus (STG), left middle/inferior frontal gyrus (MFG/IFG), right precentral gyrus, right superior parietal lobule (SPL), left postcentral gyrus, and right precuneus (voxel-level FWE-corrected *P* < 0.05)

Figure 4 illustrates voxelwise decoding accuracy maps (raw classification accuracy, %) averaged across participants for descriptive purposes, while Tables 2–4 summarize the results of the second-level statistical analyses.

**Table 2:** Brain regions showing significant decoding effects across five emotions

average of full factorial design: factor: 2 (subject group) × 2 (Asian Western vocalization) × 5 emotions				
Brain regions	MNI coordinate	BA	F	p
**L STG**	**(−50, −15, 2)**	**22**	**69.7**	**<0.001**
**R STG**	**(51, −14, 2)**	**22**	**64.3**	**<0.001**
**L IFG**	**(−42, 35, 11)**	**46**	**38.2**	**<0.001**
**L MFG**	**(−5, 8, 44)**	**32**	**33.9**	**<0.001**
**R precentral gyrus**	**(36, 11, 33)**	**9**	**31.4**	**<0.001**
**L MFG**	**(0, 50, 26)**	**9**	**29.7**	**<0.001**
**L IFG**	**(−48, 11, 20)**	**44**	**28.9**	**0.001**
**R SPL**	**(−24, −51, 48)**	**7**	**25.5**	**0.003**
**L postcentral gyrus**	**(−42, −23, 45)**	**2**	**23.1**	**0.009**
**R precuneus**	**(27, −68, 32)**	**7**	**22.6**	**0.011**
**R SFL**	**(15, 51, 21)**	**9**	**21.8**	**0.015**
**L precuneus**	**(−6, −60, 32)**	**7**	**21.2**	**0.020**
**ACC**	**(−2, 29, 30)**	**32**	**20.3**	**0.029**

Full-factorial design: 2 (Group: Asian, Western) × 2 (Stimulus: TAV, MAV) × 5 (Emotion).

Peak-level FWE-corrected *P* < 0.05.

**Table 3:** Brain regions showing a significant main effect of emotion in multivariate pattern analysis (MVPA)

**Brain regions**	**MNI coordinate**	**BA**	**F**	**p**	**Angry**	**90%CI**	**Sad**	**90%CI**	**Neutral**	**90%CI**	**Happy**	**90%CI**	**Pleased**	**90%CI**
**R STG**	**(51, −11, 0)**	**22**	**70.4**	**<0.001**	**16.57**	**[15.54–17.60]**	**31.13**	**[30.09–32.18]**	**19.35**	**[18.30–20.40]**	**20.20**	**[19.16–21.24]**	**22.58**	**[21.44–23.73]**
**L insula**	**(−51, −21, 14)**	**40**	**69.6**	**<0.001**	**17.70**	**[16.80–18.59]**	**29.62**	**[28.72–30.53]**	**19.2**	**[18.29–20.11]**	**19.12**	**[18.22–20.02]**	**21.44**	**[20.44–22.43]**
**R pMTG**	**(32, −57, 30)**	**39**	**32.0**	**<0.001**	**17.95**	**[17.11–18.79]**	**24.46**	**[23.61–25.31]**	**18.44**	**[17.58–19.30]**	**18.65**	**[17.80–19.49]**	**23.36**	**[22.43–24.30]**
**R SFG**	**(6, 51, 27)**	**9**	**26.9**	**<0.001**	**18.33**	**[17.39–19.27]**	**24.85**	**[23.91–25.80]**	**18.31**	**[17.36–19.27]**	**19.45**	**[18.51–20.39]**	**23.92**	**[22.87–24.96]**
**L ACC**	**(−6, 8, 42)**	**32**	**25.0**	**<0.001**	**18.65**	**[17.72–19.58]**	**24.57**	**[23.63–25.61]**	**19.46**	**[18.51–20.41]**	**19.06**	**[18.13–20.00]**	**24.55**	**[23.52–25.58]**
**R MFG**	**(41 11 29)**	**9**	**19.7**	**<0.001**	**18.09**	**[17.13–19.05]**	**24.75**	**[23.78–25.72]**	**19.81**	**[18.84–20.79]**	**19.31**	**[18.34–20.27]**	**22.74**	**[21.68–23.81]**

Reported values represent peak coordinates (MNI x, y, z) and raw decoding accuracy (%).

Voxel-level FWE-corrected *P* < 0.05.

**Table 4:** Percentages of significant brain areas in each emotional category for the main effect of emotion

main effect of 5 emotions: 2 × 2 × 5 full factorial design						
Emotion/Brain regions	MNI coordinate	BA	F	p		90%CI
**angry/L STG**	**(−36, −30, 14)**	**41**	**44.7**	**<0.001**	**15.14**	**13.95–16.34**
**angry/R insula**	**(42, −21, 11)**	**13**	**42.7**	**<0.001**	**15.40**	**14.25–16.56**
**angry/L STG**	**(−47, −6, −11)**	**38**	**30.0**	**<0.001**	**17.01**	**16.11–17.91**
**sad/R STG**	**(51, −11, 0)**	**22**	**308.0**	**<0.001**	**33.92**	**32.61–35.22**
**sad/L HG**	**(−53, −20, 12)**	**41**	**306.9**	**<0.001**	**32.46**	**31.29–33.63**
**sad/R precuneus**	**(24, −75, 15)**	**31**	**79.2**	**<0.001**	**26.54**	**25.33–27.74**
**sad/R SFG**	**(14, 51, 27)**	**9**	**77.7**	**<0.001**	**26.53**	**25.32–27.75**
**sad/R MFG**	**(39, 11, 29)**	**9**	**68.9**	**<0.001**	**25.94**	**24.76–27.11**
**sad/L ACC**	**(−5, 9, 38)**	**32**	**68.6**	**<0.001**	**25.77**	**24.62–26.91**
**sad/L cuneus**	**(−5, −72, 21)**	**18**	**48.9**	**<0.001**	**25.36**	**24.10–26.62**
**neutral/L STG**	**(−42–39 14)**	**41**	**19.1**	**0.049**	**16.72**	**15.49–17.96**
**pleased/L precuneus**	**(−6, −59, 32)**	**7**	**64.7**	**<0.001**	**26.83**	**25.43–28.23**
**pleased/L MFG**	**(0, 32, 35)**	**6**	**60.9**	**<0.001**	**27.15**	**25.64–28.66**
**pleased/L MFG**	**(−5, 8, 44)**	**32**	**58.5**	**<0.001**	**25.97**	**24.65–27.29**
**pleased/L IFG**	**(−44, 30, 12)**	**46**	**58.4**	**<0.001**	**26.61**	**25.19–28.04**
**pleased/L SMG**	**(−45–50 33)**	**40**	**53.6**	**<0.001**	**25.76**	**24.46–27.05**
**pleased/R IPL**	**(38–50 39)**	**39**	**48.3**	**<0.001**	**25.60**	**24.27–26.93**

Bar values correspond to mean ± 90% confidence intervals of raw decoding accuracy (%). Voxel-level FWE-corrected *P* < 0.05.

**Figure 5:**
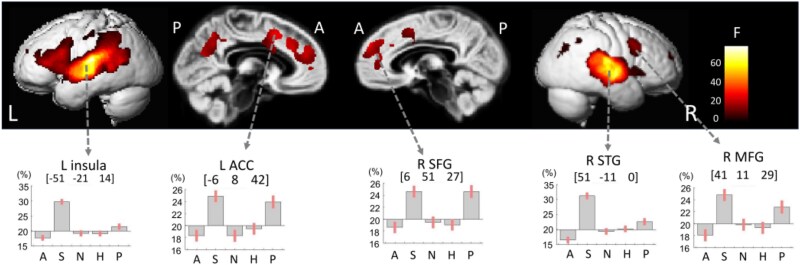
Brain regions showing the main effect of emotion in MVPA. Significant clusters were identified in the right superior temporal gyrus (STG), left insula, posterior right middle temporal gyrus (MTG), right superior frontal gyrus (SFG), left anterior cingulate cortex (ACC), and right middle frontal gyrus (MFG) (voxel-level FWE-corrected *P* < 0.05). Bar graphs show mean ± 90% confidence intervals of raw decoding accuracy (%) for the five emotion categories

A significant main effect of Emotion was observed in several cortical and subcortical regions, including the bilateral anterior cingulate cortex (ACC), inferior frontal gyrus (IFG), and insula (p < 0.05, voxel-level family-wise error [FWE] corrected; see Fig. 5, Table 3).

In the bilateral STG, one-sample MVPA analyses revealed robust emotion-specific decoding accuracy that reached statistical significance at the group level (p < 0.05, voxel-level FWE-corrected).

However, left STG did not show a significant main effect of Emotion in the factorial ANOVA and is therefore not included among the regions reported in Table 3.

No significant main effect of **Group** (Asian vs. Western participants) was detected at the whole-brain level (*p* > 0.05).

**Figure 6:**
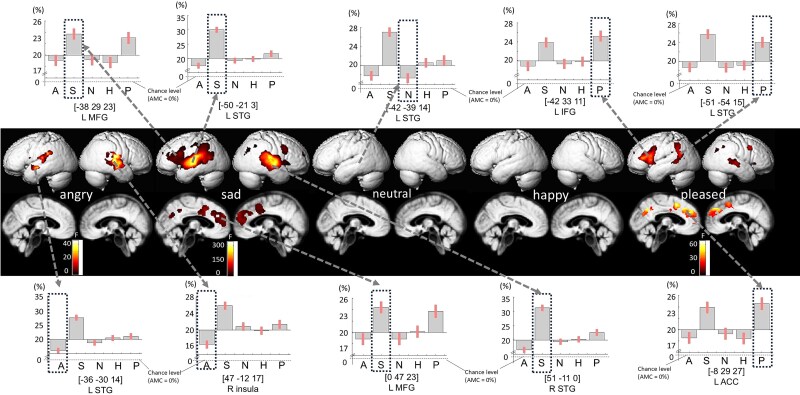
Raw decoding accuracy for each emotional category in the main effect of emotion. Bars represent mean raw decoding accuracy across participants, with error bars indicating 90% confidence intervals. The dashed horizontal line indicates the theoretical chance level (20%) for five-class classification

A significant main effect of **Stimulus Type** (TAV vs. MAV) was found in the bilateral STG and left superior temporal sulcus (STS) (p < 0.05, voxel-level FWE corrected; Fig. 6, Table 4).

Decoding accuracy was generally higher for TAV than for MAV voices, particularly within the left STG and insula.

In addition, a significant **Emotion × Stimulus** interaction emerged in the left STG (p < 0.05, voxel-level FWE corrected), where Neutral and Happy voices were decoded more accurately for TAV than MAV, whereas Sad and Angry voices showed the opposite pattern.

No significant Emotion × Group or three-way Emotion × Group × Stimulus interaction was observed (p > 0.05).

### Emotion-dependent decoding patterns

Emotion-dependent decoding patterns were observed in several cortical and subcortical regions, including the right superior temporal gyrus (STG), left insula, posterior right middle temporal gyrus (pMTG), right superior frontal gyrus (SFG), left anterior cingulate cortex (ACC), and right middle frontal gyrus (MFG) (p < 0.05, peak-level FWE-corrected; see Figs. 5–6 and Tables 3–4).

For the left STG, raw decoding accuracies (mean [90% CI]) were: Angry 16.57% [15.54–17.60], Sad 31.13% [30.09–32.18], Neutral 19.35% [18.30–20.40], Happy 20.20% [19.16–21.24], and Pleased 22.58% [21.44–23.73].

For the right STG, raw decoding accuracies were: Angry 17.70% [16.80–18.59], Sad 29.62% [28.72–30.53], Neutral 19.20% [18.29–20.11], Happy 19.12% [18.22–20.02], and Pleased 21.44% [20.44–22.43].

Robust emotion-dependent modulation was observed, with Sad voices showing the highest decoding accuracy, followed by Pleased voices, indicating stronger distinctiveness of affective representations in auditory cortical regions.

Figure 5 and Table 4 summarize the spatial distribution and relative proportions of voxels showing significant effects for each emotion under the main effect of Emotion (p < 0.05, peak-level FWE-corrected). For Angry vocalizations, clusters were identified in the left STG (15.14% [13.95–16.34]) and right insula (15.40% [14.25–16.56]). For Sad vocalizations, the most extensive clusters were observed in the right STG (33.92% [32.61–35.22]), left Heschl’s gyrus (32.46% [31.29–33.63]), right precuneus (26.54% [25.33–27.74]), right SFG (26.53% [25.32–27.75]), right MFG (25.94% [24.76–27.11]), left ACC (25.77% [24.62–26.91]), and left cuneus (25.36% [24.10–26.62]). For Neutral vocalizations, a cluster was identified in the left STG with comparatively modest decoding accuracy. For Pleased vocalizations, clusters were observed in the left precuneus (26.83% [25.43–28.23]), left MFG (27.15% [25.64–28.66]), left inferior frontal gyrus (26.61% [25.19–28.04]), left supramarginal gyrus (25.76% [24.46–27.05]), and right inferior parietal lobule (25.60% [24.47–26.93]).

Overall, these results demonstrate that patterns of raw decoding accuracy varied systematically with emotional category, with Sad and Pleased voices showing the strongest differentiation within bilateral auditory and higher-order association cortices.

### Emotion × stimulus interaction

**Figure 7:**
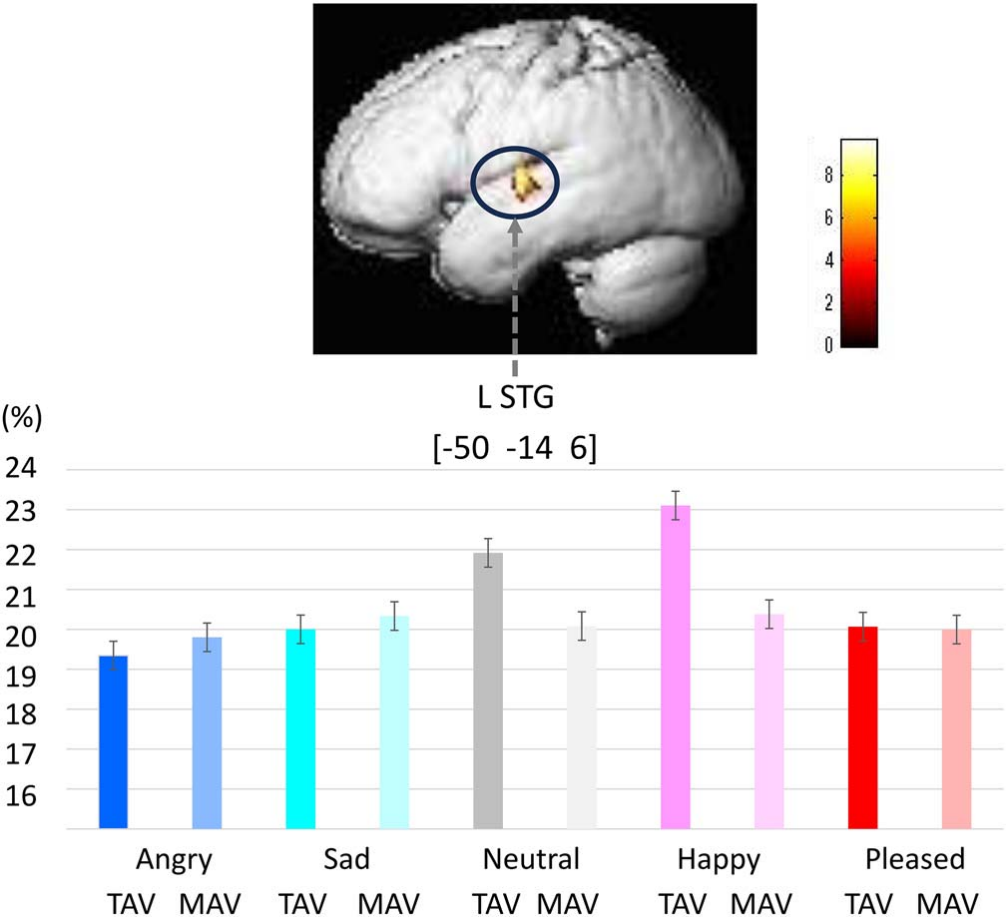
Interaction between emotion and stimulus type (TAV–MAV). A significant emotion × stimulus interaction was observed in the left superior temporal gyrus (STG) (voxel-level FWE-corrected *P* < 0.05). Raw decoding accuracy was higher for TAV than MAV in neutral and happy voices, suggesting stimulus-set–dependent modulation of auditory representations

Figure 7 and Table 5 illustrate the significant interaction effect between **Emotion and Stimulus Type** (TAV–MAV) at the whole-brain level (*P* < 0.05, peak-level FWE-corrected).

**Table 5:** Interaction between emotion and stimulus type (TAV–MAV)

Interaction effect between TAVMAV × emotions				
TAVMAV Emotion/Brain regions	MNI coordinate	BA	F	p
**L STG**	**(−50, −16 6)**	**22**	**9.3**	**<0.001**
	**percentage**	**90%CI**		
**TAV angry**	**19.35**	**19.02–19.67**		
**MAV angry**	**19.80**	**19.48–20.13**		
**TAV sad**	**20.00**	**19.68–20.32**		
**MAV sad**	**20.33**	**20.01–20.66**		
**TAV neutral**	**21.92**	**21.59–22.24**		
**MAV neutral**	**20.08**	**19.75–20.41**		
**TAV happy**	**23.10**	**22.78–23.43**		
**MAV happy**	**20.38**	**20.04–20.72**		
**TAV pleased**	**20.07**	**19.74–20.39**		
**MAV pleased**	**20.00**	**19.64–20.36**		

The left superior temporal gyrus (STG) showed a significant Emotion × Stimulus interaction (voxel-level FWE-corrected *P* < 0.05). Reported values correspond to raw decoding accuracy (%) for TAV and MAV stimuli in each emotion.

A significant Emotion × Stimulus interaction was observed in the left superior temporal gyrus (STG), where raw decoding accuracy differed between the two vocal sets.

Specifically, raw decoding accuracies (%) were higher for TAV than for MAV in *Neutral* and *Happy* voices (see Fig. 7):

*Neutral* – TAV 21.92% [21.59–22.24], MAV 20.08% [19.75–20.41];

*Happy* – TAV 23.10% [22.78–23.43], MAV 20.38% [20.04–20.72].

In contrast, raw decoding accuracies did not significantly differ between TAV and MAV for *Angry*, *Sad*, and *Pleased* voices (all p > 0.05).

These results are consistent with stimulus-set–dependent modulation of auditory representations in the left STG, whereby Japanese (TAV) emotional voices elicited slightly higher decoding accuracy than Canadian (MAV) voices for specific emotion categories.

## Discussion

The present fMRI study investigated how cultural context modulates neural representations of emotional voice perception, using the Tokyo Affective Voices (TAV) and the Montreal Affective Voices (MAV) (Belin et al. 2008; Koeda et al. 2013). We aimed to clarify the neural mechanisms underlying auditory affective processing in healthy individuals as a foundation for future translational applications in psychiatry (Schirmer and Kotz 2006; Fecteau et al. 2004). By applying multivariate pattern analysis (MVPA), we identified distributed cortical patterns that reliably decoded emotional categories from nonverbal vocalizations (Haynes and Rees 2006; Norman et al. 2006). Across both Asian and Western participants, significant decoding accuracy was observed in bilateral superior temporal gyri (STG), inferior and middle frontal gyri, anterior cingulate cortex (ACC), and insula—regions consistently implicated in auditory–emotional processing (Schirmer and Kotz 2006; Fecteau et al. 2004). Among the five emotions examined, sadness elicited the most robust and distinctive decoding accuracy, whereas happiness and pleasure produced more variable patterns across individuals and cultural contexts (Juslin and Laukka 2003; Pell et al. 2009). These results support the notion that negative emotions such as sadness are represented in more stable and convergent neural codes, while positive emotions involve more flexible and context-dependent representations (Cowen et al. 2021; Elfenbein and Ambady 2002).

Cultural effects were modest but detectable: decoding accuracies for neutral and happy voices were slightly higher for TAV than for MAV, suggesting subtle tuning of the left STG to culturally congruent emotional cues (Koeda et al. 2013; Sauter et al. 2010). These findings bridge behavioral and neural dimensions of emotional voice processing and underscore how MVPA can reveal fine-grained cultural modulations in distributed brain representations (Kriegeskorte et al. 2006; Haxby et al. 2001).

Importantly, this framework may contribute to psychiatric research by informing the characterization of emotional processing based on distributed neural patterns. Disorders such as depression and schizophrenia are characterized by altered perception and expression of affective prosody, particularly for sadness and pleasure (Edwards et al. 2002; Pinkham et al. 2020). Identifying stable neural signatures of these emotions in healthy individuals provides a benchmark for evaluating dysfunction in patient populations (Schirmer and Kotz 2006; Cotter et al. 2018). Integrating culturally validated stimuli such as TAV and MAV with multivariate neuroimaging approaches may thus support future efforts aimed at identifying objective markers relevant to emotional dysregulation, while avoiding claims of immediate diagnostic application (Cowen et al. 2021; Belin et al. 2008).

### Universal neural mechanisms of vocal emotion recognition

Our study investigated how the human brain decodes emotional meaning from nonverbal vocalizations across cultural contexts (Belin et al. 2008; Koeda et al. 2013). Consistent with our hypothesis, sadness showed the most stable and accurate decoding pattern, with high classification performance within bilateral superior temporal gyri (STG) and left inferior frontal gyrus (IFG) (Juslin and Laukka 2003; Pell et al. 2009). These regions are key components of the auditory–emotional network that integrates acoustic and affective cues into categorical representations (Schirmer and Kotz 2006; Belin et al. 2008). Their robust activation across both Asian and Western participants suggests a universal mechanism for perceiving social distress and negative affect (Elfenbein and Ambady 2002; Sauter et al. 2010). Sadness tends to convey universally recognizable acoustic features, such as low pitch, reduced intensity, and slow temporal modulation, which are reliably processed in the STG and insula (Fecteau et al. 2004; Pell et al. 2009; Craig 2009). These findings align with prior behavioral evidence indicating that sadness and anger are consistently recognized across languages and cultures (Elfenbein and Ambady 2002; Sauter et al. 2010).

Clinically, this universal neural signature of sadness may provide an essential reference for understanding emotional dysregulation in psychiatric conditions (Schirmer and Kotz 2006; Cotter et al. 2018). Patients with depression often exhibit blunted activation within the STG and IFG when processing negative emotions, reflecting reduced affective resonance (Schirmer and Kotz 2006; Cotter et al. 2018; Frodl et al. 2008). Similarly, schizophrenia is associated with impaired decoding of prosodic cues, particularly in left temporal–frontal pathways implicated here (Edwards et al. 2002; Pinkham et al. 2020). The strong and reproducible neural pattern for sadness observed in healthy individuals may therefore serve as a normative benchmark for assessing abnormal emotional coding in clinical populations (Schirmer and Kotz 2006; Pell et al. 2009). Taken together, our findings confirm the first part of our hypothesis and support the idea that negative emotions, especially sadness, engage stable, universal brain mechanisms of auditory emotion perception (Cowen et al. 2021; Elfenbein and Ambady 2002).

### Culture-specific tuning and TAV-dominant emotional representations

Our second hypothesis predicted modest cultural effects, with culturally congruent stimuli (TAV for Asians, MAV for Western participants) showing slightly higher decoding accuracy (Elfenbein and Ambady 2002; Koeda et al. 2013). This was partially confirmed: decoding accuracies for neutral and happy voices were higher for TAV than MAV, whereas no reliable group effect was found (Koeda et al. 2013; Tanaka et al. 2010). These results suggest subtle stimulus-set–dependent modulation of the left STG toward familiar prosodic contours, rather than large-scale network differences (Schirmer and Kotz 2006; Tanaka et al. 2010).

Prior research has shown that positive emotions are more sensitive to sociocultural display rules and acoustic conventions, while negative emotions are more universally encoded (Matsumoto and Ekman 1989; Scherer et al. 2001). The current data extend this view by revealing that cultural familiarity enhances decoding stability primarily for positive or ambiguous emotions that rely heavily on contextual interpretation (Cowen et al. 2021; Sauter et al. 2010). The modest TAV advantage may reflect higher harmonic-to-noise ratios and shorter durations in Japanese affective speech (Koeda et al. 2013), consistent with culturally restrained expression of positive affect (Matsumoto and Ekman 1989).

From a clinical perspective, these cultural modulations illustrate how social learning and emotional display rules shape the neural decoding of vocal affect (Cotter et al. 2018; Edwards et al. 2002). In psychiatry, such mechanisms may influence how individuals interpret others’ emotional intentions, particularly in multicultural or high-stress environments (Edwards et al. 2002; Min and Schirmer 2011). Patients with mood or anxiety disorders often misinterpret neutral or positive tones as negative or threatening (Cotter et al. 2018). Understanding how cultural familiarity modulates auditory emotion perception could therefore inform more nuanced approaches to cross-cultural mental health research and practice (Paulmann and Uskul 2014). The current findings thus confirm that cultural tuning influences perceptual weighting rather than the fundamental architecture of the auditory–emotional network, indicating that universal and culture-specific mechanisms coexist within the same neural system (Schirmer and Kotz 2006; Scherer et al. 2001).

### Network representations centered on the auditory cortex

MVPA revealed high decoding accuracy not only in the STG but also in the left middle and inferior frontal gyri, confirming that emotional voice processing engages a distributed fronto-temporal network (Schirmer and Kotz 2006; Pell et al. 2009; Belin et al. 2000). These regions have been repeatedly identified as part of the ‘voice perception network’ responsible for integrating spectral details with emotional meaning (Pell et al. 2009; Belin et al. 2000). Importantly, while sadness was externally coherent across listeners, pleasure elicited high decoding accuracy in medial frontal and cingulate cortices despite lower behavioral accuracy (Cowen et al. 2021; Banse and Scherer 1996). This dissociation indicates that internally oriented emotions such as pleasure are represented within introspective and reward-related circuits rather than external perceptual pathways (Kriegeskorte et al. 2006; Craig 2009; Kringelbach and Berridge 2009). Thus, our findings only partially matched the prediction that pleasure would show low decoding accuracy overall—its neural representation was strong but internally localized (Cowen et al. 2021; Kriegeskorte et al. 2006; Kringelbach and Berridge 2009).

This distinction between externally shared and internally experienced emotions provides new insight into the hierarchical structure of affective processing (Schirmer and Kotz 2006; Craig 2009). The STG and IFG encode externally perceivable cues, while the anterior cingulate and medial frontal cortices represent internal emotional value (Pell et al. 2009; Rushworth et al. 2007). Such dual coding may underlie the ability to differentiate one’s own feelings from those of others, a capacity often compromised in autism spectrum disorder and dementia (Edwards et al. 2002; Pinkham et al. 2020). Degeneration or dysconnectivity within this fronto-temporal–cingulate system can produce impaired self-other distinction and emotional flattening (Edwards et al. 2002; Rushworth et al. 2007). Therefore, mapping these distributed patterns through MVPA offers a neurobiological framework for understanding how external and internal affective representations diverge across both healthy and clinical populations. This integrated model underscores that the human brain encodes pleasure and sadness through distinct yet complementary neural hierarchies (Cowen et al. 2021; Banse and Scherer 1996).

Beyond their theoretical implications, the present findings highlight the potential value of combining culturally validated emotional voice stimuli with multivariate neural decoding approaches. Rather than proposing a specific diagnostic implementation, our results outline a conceptual framework in which distributed neural patterns could contribute to more objective characterization of affective voice processing. Future work may explore how such frameworks could be extended and validated in clinical and applied settings, including psychiatric populations, while carefully addressing issues of generalizability, robustness, and ethical deployment.

### Limitations

Several limitations should be considered when interpreting the present findings.

First, the sample size was moderate and consisted exclusively of healthy young adults, limiting generalizability to older or clinical populations. Second, all vocal stimuli were produced by female actors and presented to both male and female listeners, preventing examination of potential gender interactions in emotional voice decoding. Third, the vocalizations were acted rather than spontaneous, which may have enhanced acoustic clarity but reduced ecological validity. Future work should incorporate more naturalistic and dynamic affective expressions to validate the present results. Fourth, cultural familiarity was inferred from participants’ nationality rather than objectively quantified by measures of acculturation or linguistic exposure. Finally, while our analysis revealed robust decoding patterns using multivariate pattern analysis, the cross-sectional design cannot address longitudinal changes or treatment-related modulation of emotional processing. Future longitudinal and patient-based studies integrating fMRI, behavioral indices, and clinical assessments are necessary to evaluate whether these decoding patterns can serve as reliable biomarkers of affective dysfunction.

## Conclusion

This study investigated how the human brain decodes emotional meaning from nonverbal vocalizations across cultural contexts. Using functional MRI and multivariate pattern analysis, we identified distributed neural representations underlying five emotional categories expressed in Japanese and Canadian voices. Consistent with our hypotheses, sadness exhibited the most stable and accurate decoding pattern, with strong classification within the bilateral superior temporal gyri and left inferior frontal gyrus. These regions constitute a universal auditory–emotional network that integrates acoustic features and affective meaning beyond linguistic context. In contrast, pleasure showed lower behavioral accuracy but higher neural decoding within medial prefrontal and cingulate regions, suggesting an internally oriented representation of positive affect. These results indicate that externally shared emotions such as sadness and internally experienced emotions such as pleasure are encoded through distinct yet complementary neural hierarchies. Cultural influences were modest but detectable, with slightly higher decoding accuracy for neutral and happy voices in the culturally familiar TAV stimuli. This suggests that cultural familiarity fine-tunes perceptual weighting without altering the universal architecture of the auditory–emotional network. Clinically, these findings provide a normative foundation for evaluating emotional recognition deficits observed in depression, schizophrenia, autism, and dementia. Decoding-based fMRI approaches using culturally validated stimuli such as TAV and MAV may thus contribute to future efforts aimed at identifying objective markers relevant to affective dysfunction. Together, these findings provide neurobiological evidence that decoding-based neuroimaging can bridge cultural neuroscience and psychiatry by informing culturally sensitive and more objective approaches to the study of social–emotional processing. Overall, the present results reveal how universal and culture-tuned mechanisms jointly shape emotional voice processing, bridging basic neuroscience and translational psychiatry toward more personalized, empathy-centered mental healthcare.

## Supplementary Material

Supplementary_materials_kvag001

## Data Availability

All data, analysis scripts, and neuroimaging outputs supporting the findings of this study have been archived on the Open Science Framework (OSF) https://osf.io/vb6yu. The repository is currently under embargo to maintain the anonymity of peer review. All materials will be made publicly available upon publication, at which time a DOI will be provided.
