# Supplementary material for: Universal and culture-tuned neural codes for vocal emotion: an fMRI MVPA study using Japanese and Canadian voices
Source: Oxf Open Neurosci. 2026 Mar 17;5:kvag001. doi: 10.1093/oons/kvag001 (PMC13089443; doi:10.1093/oons/kvag001)
Supplement: Supplementary_materials_kvag001 [file supplementary_materials_kvag001.zip › supplement1.docx]

**Supplement Table 1**

**Definition of Emotions**

**Happy**

Joyful, glad, delighted

**Pleasure**

Amusement, diversion, or worldly enjoyment

**Angry**

Furious, very annoyed, irritable

**Disgust**

Strong distaste; loathing; repugnance; strong aversion; strong feeling of not liking something

**Fear**

Frightened, scared.

**Sad**

Unhappy, feeling down.

**Neutral**

Non-emotional feeling
