# Supplementary figures and images for: Universal and culture-tuned neural codes for vocal emotion: an fMRI MVPA study using Japanese and Canadian voices
Source: Oxf Open Neurosci. 2026 Mar 17;5:kvag001. doi: 10.1093/oons/kvag001 (PMC13089443; doi:10.1093/oons/kvag001)

Supplement Table 2


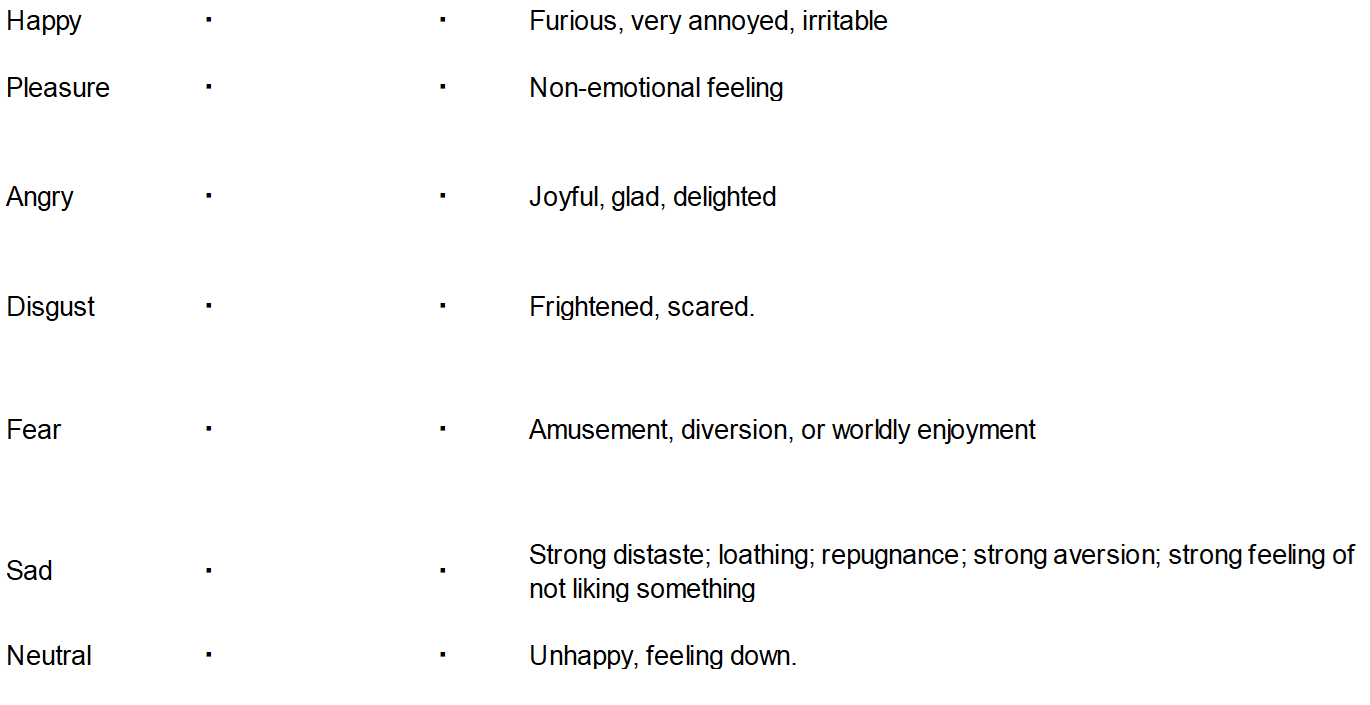

Supplement: Supplementary_materials_kvag001 [file supplementary_materials_kvag001.zip › supplement2.docx]
