# Supplementary material for: Universal and culture-tuned neural codes for vocal emotion: an fMRI MVPA study using Japanese and Canadian voices
Source: Oxf Open Neurosci. 2026 Mar 17;5:kvag001. doi: 10.1093/oons/kvag001 (PMC13089443; doi:10.1093/oons/kvag001)
Supplement: Supplementary_materials_kvag001 [file supplementary_materials_kvag001.zip › Supplemental file for export 1.docx]

**First round of review**

22-Dec-2025

Dear Dr. Koeda,

Thank you for submitting manuscript ID OXFNSC-2025-004 entitled "Universal and Culture-Tuned Neural Codes for Vocal Emotion: An fMRI MVPA Study Using Japanese and Canadian Voices" to Oxford Open Neuroscience.

Your manuscript has been evaluated by one reviewer with relevant expertise and I have read it myself. Reviewer comments can be seen below. The reviewer found your manuscript to be of interest, and I agree. Therefore, I am inviting you to submit a revision that responds to their comments.

The reviewer comments are clear so I will not list them again here. Please provide your response to all of these comments. I agree with the reviewer that it could be useful to report additional cross-classification analyses, but I will not make this a condition for acceptance.

Along with the reviewer's comments, please also add the following clarifications:

Page 3, line 56 - "The mean years of education were 16.0 ± 0.8 for the Asian group and 15.6 ± 0.6 for the
Caucasian group, with no significant difference between them". Please report details of the statistical test here.

Page 7, line 23 - "For each participant, voxelwise activation patterns from preprocessed BOLD images
were analyzed using a linear support vector machine (SVM; (Cortes and Vapnik, 1995))
classifier within spherical searchlights (radius = 8 mm)." Please report further details of how your MVPA analyses were conducted, so that these analyses could be replicated. E.g. did you use a particular toolbox or your own code? If you used your own code, what SVM implementation did you use and how did you perform multiple-class classification across five emotion categories?

Please note that this journal operates with transparent peer review. This means that if your submission is accepted for publication, the full peer review history of your article will publish online alongside your article. This includes reviewer comments, editor decision letters, and your author responses.

I invite you to respond to the reviewer(s)' comments and revise your manuscript within the next 60 days from today date. Please let me know if you would like an extension to this.

To revise your manuscript, log into https://mc.manuscriptcentral.com/oxfnsc and enter your Author Centre, where you will find your manuscript title listed under "Manuscripts with Decisions."  Under "Actions," click on "Create a Revision."  Your manuscript number has been appended to denote a revision.

You may also click the below link to start the revision process (or continue the process if you have already started your revision) for your manuscript. If you use the below link you will not be required to login to ScholarOne Manuscripts.

*** PLEASE NOTE: This is a two-step process. After clicking on the link, you will be directed to a webpage to confirm. ***

https://mc.manuscriptcentral.com/oxfnsc?URL_MASK=b36089f414d648a0868b8594aa94b748

You will be unable to make your revisions on the originally submitted version of the manuscript.  Instead, revise your manuscript using a word processing program and save it on your computer.  Please also highlight the changes to your manuscript within the document by using the track changes mode in MS Word or by using bold or colored text.

Once the revised manuscript is prepared, you can upload it and submit it through your Author Centre.

When submitting your revised manuscript, you will be able to respond to the comments made by the reviewer(s) in the space provided.  You can use this space to document any changes you make to the original manuscript.  In order to expedite the processing of the revised manuscript, please be as specific as possible in your response to the reviewer(s).

IMPORTANT:  Your original files are available to you when you upload your revised manuscript.  Please delete any redundant files before completing the submission.

Because we are trying to facilitate timely publication of manuscripts submitted to the Oxford Open Neuroscience, your revised manuscript should be uploaded within the next 60 days from today date.  If it is not possible for you to submit your revision in a reasonable amount of time, we may have to consider your paper as a new submission.

Once again, thank you for submitting your manuscript to the Oxford Open Neuroscience and I look forward to receiving your revision.

Sincerely,
Professor Sam Gilbert
Senior Editor, Oxford Open Neuroscience
[sam.gilbert@ucl.ac.uk](mailto:sam.gilbert@ucl.ac.uk)

**Response to the Editor and Reviewer**

We thank the editor for the careful evaluation of our manuscript and for the constructive suggestions.

(1) Demographic statistics (Page 3, line 56)
As requested, we have added full details of the statistical tests used to compare demographic variables between groups. Specifically, we now report Welch’s two-sample t-tests for years of education and the Edinburgh Handedness Inventory (EHI), including t values, degrees of freedom, and p values (Page 3, line 56).

(2) MVPA methodological details (Page 7, line 23)
As requested, we have added further details of the MVPA implementation to facilitate reproducibility, including clarification of the multi-class SVM approach and the software environment used for the searchlight analyses (Section 2.7). These clarifications do not change the analysis pipeline or results.

Responses to Reviewer 1

We thank the reviewer for the thoughtful and detailed comments, which have helped us to improve the clarity and rigor of the manuscript. Below we respond to each point.

1) Cross-classification
We agree that cross-classification can provide a stronger test of abstraction and generalizability. However, as also noted by the editor, additional cross-classification analyses were not required for acceptance. In this revision, we therefore did not add new cross-classification analyses, and instead focused on clarifying the scope of our conclusions and strengthening methodological transparency.

2) Cultural interpretation and absence of Group differences
We have clarified throughout the manuscript that no reliable whole-brain Group differences were observed between Asian and Western participants. In addition, we have tempered the cultural interpretation of the Emotion × Stimulus interaction by describing it more conservatively as stimulus-set–dependent modulation, consistent with the fact that acoustic and recording differences between stimulus sets may also contribute to decoding differences (see Discussion, Section 4.2).

3) Engagement with the literature on vocal emotion decoding
We appreciate this suggestion. The revised Discussion better situates our findings within the established literature on neural processing and decoding of vocal emotions/prosody, including work on voice-sensitive cortices and distributed fronto-temporal networks, while maintaining the original focus of the manuscript.

4) Correlation analysis thresholding
We agree that the correlation analysis was performed at an uncorrected threshold (p = 0.001, cluster extent ≥ 20 voxels). We have ensured that the interpretation is appropriately cautious and clearly states this thresholding approach (Results/Discussion).

Minor points and clarifications
- We acknowledge that second-level inference on classification accuracies using parametric tests has limitations and have ensured the corresponding wording is appropriately cautious.
- We revised wording where needed to avoid over-interpretation based on reverse inference, and we now present such interpretations as tentative.
- We improved clarity and consistency of terminology by replacing “Caucasian” with “Western” throughout the manuscript, figures, and figure legends. This includes updating labels in Figures 1–3 and harmonizing Group terminology in the text and legends.
- Figure and legend consistency has been checked and corrected where applicable.
- Added demographic test statistics for years of education and EHI (Welch’s t-tests) (Page 3, line 56).


Reviewer: 1

Comments to the Author
This manuscript examines the neural processing of non-verbal emotional vocalizations using multivariate pattern analysis of fMRI data, with a particular focus on cross-cultural differences between Asian (N = 22) and Western (N = 19) listeners. The study contrasts responses to the recently introduced “Tokyo Affective Voices” with those to the well-established “Montreal Affective Voices,” providing a valuable opportunity to assess cultural influences on vocal emotion decoding. The topic is timely and important, and the overall study design is sound. Below, I outline several ways in which the interpretation of the findings could be strengthened, and where additional analyses or clarifications would provide more compelling support for the authors’ claims.

- The manuscript aims to identify “universal” and culture-specific neural codes by comparing decoding performance for Japanese and Canadian speakers in Asian and Western listeners. However, a stronger test of shared versus distinct neural representations would involve explicit cross-classification (Kaplan et al., 2015). For instance, classifiers could be trained on Japanese speakers and tested on Canadian speakers (and vice versa) or trained on Asian participants and tested on Western participants (and vice versa). If the authors wish to argue for “universal” neural codes, in the context of this study meaning codes that generalize across Japanese and Canadian cultures, this should be demonstrated through cross-speaker and cross-listener generalizability rather than between-group differences (or their absence) in decoding alone.
- The manuscript should state more clearly that no group differences were observed between Asian and Western listeners. While this is not problematic in itself, the paper still invokes “cultural effects” based on decoding differences between stimulus sets (Tokyo vs. Montreal Affective Voices). In my view, these differences do not support cultural interpretations, as they most likely reflect acoustic differences between the respective stimuli rather than culture-specific neural coding.
- The manuscript does not sufficiently engage with the substantial literature on vocal emotion decoding. Several prior studies have used multivariate pattern analysis to investigate vocal emotion representations (Ethofer et al., 2009; Kotz et al., 2013; Leipold et al., 2023), yet these are not discussed. More broadly, the findings are not well integrated into existing work on neural vocal emotion processing. I encourage the authors to situate their results within this body of work, for example reviewed in Frühholz & Ceravolo (2018).
- The correlation analysis was conducted without family-wise error correction (initial threshold p = 0.001 uncorrected, cluster size ≥ 20 voxels). The interpretation of this result should be tempered accordingly.

Minor
- I am not suggesting that the authors re-run their decoding analyses, but it would be important to acknowledge that relying on parametric ANOVAs and t-tests for second-level inference on classification accuracies is not ideal (Allefeld et al., 2016; Stelzer et al., 2013).
- On page 3, L21: The paper uses “stable representations" as reflecting higher decoding accuracy. However, I would argue that stable representations are those that would show higher reliability in a longitudinal setting.
- The reported correlations between individual differences in neural decoding and behavioral performance do not, in my view, justify the conclusion that “sensorimotor-affective integration supports perceptual discrimination.”
- The characterization of sadness as an externally oriented emotion and pleasure as an internally oriented emotion appears speculative, particularly because it is derived from reverse inference based on decoding in specific brain regions (e.g., medial prefrontal cortex and cingulate for pleasure). This interpretation should be explicitly presented as tentative rather than as a definitive claim.
- The manuscript highlights psychiatric disorders such as schizophrenia as a key clinical context but mentions autism only in passing, despite substantial evidence that atypical emotion processing, particularly for sadness (Boraston et al., 2007), is a prominent feature of autism. The framing would be strengthened by engaging more with the extensive literature on emotional (voice) processing and recognition in autism.
- The manuscript should specify precisely what the statistical maps in Figure 4 represent. Do these maps reflect an omnibus test across all Group × Stimulus × Emotion combinations?
- In Fig. 3b, the labels are likely wrong as they mention TAV Caucasian twice and miss the MAV Caucasian category.
- Consistency in the use of “Western” versus “Caucasian” would improve clarity and readability.

References:

Allefeld, C., Görgen, K., & Haynes, J.-D. (2016). Valid population inference for information-based imaging: From the second-level t-test to prevalence inference. NeuroImage, 141, 378–392. https://doi.org/10.1016/j.neuroimage.2016.07.040
Boraston, Z., Blakemore, S.-J., Chilvers, R., & Skuse, D. (2007). Impaired sadness recognition is linked to social interaction deficit in autism. Neuropsychologia, 45(7), 1501–1510. https://doi.org/10.1016/j.neuropsychologia.2006.11.010
Ethofer, T., Van De Ville, D., Scherer, K., & Vuilleumier, P. (2009). Decoding of Emotional Information in Voice-Sensitive Cortices. Current Biology, 19(12), 1028–1033. https://doi.org/10.1016/j.cub.2009.04.054
Frühholz, S., & Ceravolo, L. (2018). The Neural Network Underlying the Processing of Affective Vocalizations. In S. Frühholz & P. Belin (Eds.), The Oxford Handbook of Voice Perception. https://doi.org/10.1093/oxfordhb/9780198743187.013.19
Kaplan, J. T., Man, K., & Greening, S. G. (2015). Multivariate cross-classification: Applying machine learning techniques to characterize abstraction in neural representations. Frontiers in Human Neuroscience, 9. https://www.frontiersin.org/article/10.3389/fnhum.2015.00151
Kotz, S. A., Kalberlah, C., Bahlmann, J., Friederici, A. D., & Haynes, J.-D. (2013). Predicting vocal emotion expressions from the human brain. Human Brain Mapping, 34(8), 1971–1981. https://doi.org/10.1002/hbm.22041
Leipold, S., Abrams, D. A., Karraker, S., & Menon, V. (2023). Neural decoding of emotional prosody in voice-sensitive auditory cortex predicts social communication abilities in children. Cerebral Cortex, bhac095. https://doi.org/10.1093/cercor/bhac095
Stelzer, J., Chen, Y., & Turner, R. (2013). Statistical inference and multiple testing correction in classification-based multi-voxel pattern analysis (MVPA): Random permutations and cluster size control. NeuroImage, 65, 69–82. https://doi.org/10.1016/j.neuroimage.2012.09.063

Associate Editor
Comments to the Author:
(There are no comments.)

**Second round of review**

**3-Jan-2026**

Dear Dr. Koeda,

Thank you for submitting your revision, OXFNSC-2025-004.R1 entitled "Universal and Culture-Tuned Neural Codes for Vocal Emotion: An fMRI MVPA Study Using Japanese and Canadian Voices".

I have considered this alongside the previous reviewer comments and I'm grateful for your revisions. However, on reading the article I encountered some difficulties understanding the methods you presented and the interpretation of some of the results.

In order to consider your manuscript further, please address the following points and carefully review the statistical reporting throughout.

p. 7 - You say that MVPA was implemented via in-house MATLAB scripts but you do not provide further detail. At a minimum, please specify which SVM implementation you used (e.g. libsvm) and whether you used default parameters.

p. 10 - you say “In the bilateral STG, multivariate pattern analysis (MVPA) revealed distinct emotion-specific decoding accuracies”. Is this the same thing as saying there is a main effect of Emotion? If so, why is left STG not amongst the regions listed in the previous paragraph where you list “several cortical and subcortical regions” showing a significant main effect of emotion, and why is it not included in Table 3?

p. 11 - “For Neutral vocalizations, classification accuracy in the left STG (16.72% [15.49-17.96]) was significantly above chance.” How is 16.72% greater than the chance decoding level of 20%? At first I assumed you were reporting the chance-corrected decoding accuracies, but this does not seem to be the case because your figures imply a chance level of 20%. Please clarify.

Figure 8 - Correlation between behavioral accuracy and neural decoding accuracy. Please elaborate on exactly how this analysis was performed. From the figure, it seems that the effect is driven just as much by participants with below-chance as above-chance accuracy. It is hard to understand how individual variation in below-chance accuracy levels can be meaningful. This, coupled with the implausible near-perfect correlation between the neural and behavioural measure, suggests a statistical artifact. Please either remove this analysis of provide a clearer justification that it is robust.

Please note that this journal operates with transparent peer review. This means that if your submission is accepted for publication, the full peer review history of your article will publish online alongside your article. This includes reviewer comments, editor decision letters, and your author responses.

To revise your manuscript, log into https://mc.manuscriptcentral.com/oxfnsc and enter your Author Centre, where you will find your manuscript title listed under "Manuscripts with Decisions."  Under "Actions," click on "Create a Revision."  Your manuscript number has been appended to denote a revision.

You may also click the below link to start the revision process (or continue the process if you have already started your revision) for your manuscript. If you use the below link you will not be required to login to ScholarOne Manuscripts.

*** PLEASE NOTE: This is a two-step process. After clicking on the link, you will be directed to a webpage to confirm. ***

https://mc.manuscriptcentral.com/oxfnsc?URL_MASK=5a6e689449224e8394cfdd904f4b3b66

You will be unable to make your revisions on the originally submitted version of the manuscript.  Instead, revise your manuscript using a word processing program and save it on your computer.  Please also highlight the changes to your manuscript within the document by using the track changes mode in MS Word or by using bold or colored text.

Once the revised manuscript is prepared, you can upload it and submit it through your Author Centre.

When submitting your revised manuscript, you will be able to respond to the comments made by the reviewer(s) in the space provided.  You can use this space to document any changes you make to the original manuscript.  In order to expedite the processing of the revised manuscript, please be as specific as possible in your response to the reviewer(s).

IMPORTANT:  Your original files are available to you when you upload your revised manuscript.  Please delete any redundant files before completing the submission.

Because we are trying to facilitate timely publication of manuscripts submitted to the Oxford Open Neuroscience, your revised manuscript should be uploaded within the next 60 days from todays date.  If it is not possible for you to submit your revision in a reasonable amount of time, we may have to consider your paper as a new submission.

Once again, thank you for submitting your manuscript to the Oxford Open Neuroscience and I look forward to receiving your revision.

Sincerely,
Professor Sam Gilbert
Senior Editor, Oxford Open Neuroscience
sam.gilbert@ucl.ac.uk


Associate Editor
Comments to the Author:
(There are no comments.)

**Response to the Editor and Reviewer**
Q1: Clarification of MVPA implementation

A1: We thank the editor for this important request for clarification.

In the revised manuscript, we now explicitly specify the SVM implementation used for the MVPA analyses.
MVPA was implemented using custom MATLAB scripts together with the LIBSVM library (Chang and Lin, 2011).
A linear support vector machine was used with default parameters (cost parameter C = 1), and multi-class classification across the five emotion categories was performed using a one-versus-one scheme.

These details have been added to Section 2.7 (Methods) to ensure full reproducibility of the MVPA analyses.

Q2 Clarification of STG results and main effect of Emotion

A2
We thank the editor for highlighting this important point.

We agree that our original wording was potentially confusing by conflating two distinct statistical analyses.
In the revised manuscript, we now clearly distinguish between (i) regions showing a significant main effect of Emotion in the second-level factorial ANOVA, and (ii) regions showing robust above-chance decoding accuracy based on one-sample MVPA analyses.

In the revised Results section, the bilateral superior temporal gyrus (STG) is described exclusively as showing robust emotion-dependent decoding accuracy in one-sample tests against chance level.
Left STG did not show a significant main effect of Emotion in the factorial ANOVA and is therefore not included among the regions reported in Table 3, which summarizes only ANOVA-based main effects.

We have revised the Results section accordingly to ensure that the terminology used consistently reflects the underlying statistical analyses and avoids conflating ANOVA main effects with one-sample decoding results.

Q3. Correlation analysis (former Fig. 8)

A3
We thank the editor for this important comment.

Following the editor’s suggestion, we have removed the correlation analysis between behavioral accuracy and neural decoding accuracy (former Fig. 8 and Table 6) from the revised manuscript.
As noted by the editor, this analysis was potentially difficult to interpret and not essential to the core claims of the paper.
The manuscript has been revised accordingly, and references to this analysis have been removed from the Results and Discussion sections.

**Third round of review**

30-Jan-2026

Dear Dr. Koeda,

Thank you or submitting your revised manuscript titled "Universal and Culture-Tuned Neural Codes for Vocal Emotion: An fMRI MVPA Study Using Japanese and Canadian Voices".

I’m grateful that you have clarified the description of the SVM implementation and removed the reporting of the correlation analysis.

However, the issue of how you are reporting the decoding accuracies remains confusing. On page 11 you say “the AMC in the left STG (16.72% [15.49-17.96]) was significantly greater than zero, indicating above-chance decoding performance”. This implies that the decoding figures reported in the text correspond to chance-corrected accuracies, where the chance level of accuracy has already been subtracted. In other words, you are saying the the true decoding accuracy in left STG is 36.72%, which is significantly greater than 20%.

This is hard to reconcile with Figure 6, where the y-axis now reports raw decoding accuracy, hence the dotted line at 20% which represents chance accuracy. This dotted line corresponds to a figure of 0% in the terminology I have quoted above, where anything above zero indicates above-chance performance. There are two issues here. First of all, it is confusing for the reader to understand which parts of the manuscript (like the text quoted above) report percentages that have had 20% subtracted, and which parts (like the axis of Figure 6) have not. I would recommend that you use a consistent metric throughout all of the reporting in the manuscript, i.e. either always report the raw decoding accuracy (where 20% implies chance) or always report the chance-corrected decoding accuracy (where 0% implies chance). You should not switch back and forward between the two.

The second issue is that it is hard to understand why the text reports AMC decoding of up to 33% (implying true decoding accuracy of 53%), but Figure 6 shows raw decoding accuracy of up to the mid-thirties, not mid-fifties. Furthermore, it is surprising that the tables report AMCs that cluster around 20%, which would make sense for a raw accuracy score (seeing as 20% implies chance) but would be a coincidence for AMC. I am inviting you to submit a revised manuscript that clarifies this issue. It is essential that any revision provides a clear explanation.

Last of all, your Table 2 caption says “brain regions showing above-accuracy-minus-chance” which is ungrammatical. It would be clearer to simply say “brain regions showing above-chance accuracy”.

Please note that this journal operates with transparent peer review. This means that if your submission is accepted for publication, the full peer review history of your article will publish online alongside your article. This includes reviewer comments, editor decision letters, and your author responses.

To revise your manuscript, log into https://mc.manuscriptcentral.com/oxfnsc and enter your Author Centre, where you will find your manuscript title listed under "Manuscripts with Decisions."  Under "Actions," click on "Create a Revision."  Your manuscript number has been appended to denote a revision.

You may also click the below link to start the revision process (or continue the process if you have already started your revision) for your manuscript. If you use the below link you will not be required to login to ScholarOne Manuscripts.

*** PLEASE NOTE: This is a two-step process. After clicking on the link, you will be directed to a webpage to confirm. ***

https://mc.manuscriptcentral.com/oxfnsc?URL_MASK=6a99a662fd224932a8f550cdb2939811

You will be unable to make your revisions on the originally submitted version of the manuscript.  Instead, revise your manuscript using a word processing program and save it on your computer.  Please also highlight the changes to your manuscript within the document by using the track changes mode in MS Word or by using bold or colored text.

Once the revised manuscript is prepared, you can upload it and submit it through your Author Centre.

When submitting your revised manuscript, you will be able to respond to the comments made by the reviewer(s) in the space provided.  You can use this space to document any changes you make to the original manuscript.  In order to expedite the processing of the revised manuscript, please be as specific as possible in your response to the reviewer(s).

IMPORTANT:  Your original files are available to you when you upload your revised manuscript.  Please delete any redundant files before completing the submission.

Because we are trying to facilitate timely publication of manuscripts submitted to the Oxford Open Neuroscience, your revised manuscript should be uploaded within the next 60 days from todays date.  If it is not possible for you to submit your revision in a reasonable amount of time, we may have to consider your paper as a new submission.

Once again, thank you for submitting your manuscript to the Oxford Open Neuroscience and I look forward to receiving your revision.

Sincerely,
Professor Sam Gilbert
Senior Editor, Oxford Open Neuroscience
sam.gilbert@ucl.ac.uk


Reviewer(s)' Comments to Author:

**Response to the Editor and Reviewer**

We thank the Editor for the careful re-evaluation of our revised manuscript and for the clear guidance regarding the reporting of decoding accuracies. We have fully addressed the concerns by adopting a single, consistent metric throughout the manuscript and clarifying all related text, figures, and tables.

1. Reporting of decoding accuracy (AMC vs. raw accuracy)

As recommended, we now report raw decoding accuracy (%) consistently throughout the manuscript, with the theoretical chance level defined as 20% for five-class classification.
All references to accuracy-minus-chance (AMC), values “greater than zero,” or chance-corrected interpretations have been completely removed from the text, tables, and figure legends.

Specifically:

All decoding values in the Results section are now explicitly described as raw decoding accuracy (%).

Figure 6 displays raw decoding accuracy with a dashed horizontal line indicating the chance level of 20%.

The Methods section (Section 2.7) clearly defines the decoding metric and chance level, ensuring transparency and reproducibility.

This unified reporting resolves the previous ambiguity between raw and chance-corrected accuracy and ensures full consistency across the manuscript.

2. Table 2 caption wording

We have corrected the wording of the Table 2 caption to remove the ungrammatical phrase “above-accuracy-minus-chance.”
The caption now reads “brain regions showing above-chance decoding accuracy,” which accurately reflects the results and aligns with the revised reporting framework.

We believe these revisions fully address the Editor’s concerns and substantially improve the clarity and consistency of the manuscript. We are grateful for the guidance and hope that the revised version is now suitable for further consideration.

**Fourth round of review**

16-Feb-2026

Dear Dr. Koeda,

Thank you for submitting your revised manuscript "Universal and Culture-Tuned Neural Codes for Vocal Emotion: An fMRI MVPA Study Using Japanese and Canadian Voices". 

I'm pleased to say that the main issues outlined in my previous decision letters have now been resolved. However, there are a few remaining issues that require attention before your manuscript can be formally accepted:

Abstract, lines 41-42 "Behavioural-neural correlations in the insulation and postcentral gyrus indicated sensorimotor-affective integration supporting perceptual discrimination." Seeing as you have removed the analysis of behavioural-neural correlations, this statement should also be removed.

Page 11, lines 42-43 "significant above-chance decoding accuracy was observed in the left STG (15.14% [13.95-16.34]) and right insula (15.40% [14.25-16.56])". These decoding accuracies of 15.14% and 15.40% are below the chance level of 20%, not "above-chance". Please check the figures and correct this statement.

Page 13, lines 47-52 "Moreover, correlations between behavioural recognition and MVPA accuracy...". This statement refers to an analysis that you have removed from the manuscript, so it should also be removed.

Page 18, lines 41-49 "Furthermore, significant correlations between behavioral recognition...". As above, these two sentences refer to an analysis that you have removed, so they should be removed as well.

To revise your manuscript, log into https://mc.manuscriptcentral.com/oxfnsc and enter your Author Centre, where you will find your manuscript title listed under "Manuscripts with Decisions."  Under "Actions," click on "Create a Revision."  Your manuscript number has been appended to denote a revision.

You may also click the below link to start the revision process (or continue the process if you have already started your revision) for your manuscript. If you use the below link you will not be required to login to ScholarOne Manuscripts.

*** PLEASE NOTE: This is a two-step process. After clicking on the link, you will be directed to a webpage to confirm. ***

https://mc.manuscriptcentral.com/oxfnsc?URL_MASK=0d14d29419e244b7a626cb0dc6ef0512

You will be unable to make your revisions on the originally submitted version of the manuscript.  Instead, revise your manuscript using a word processing program and save it on your computer.  Please also highlight the changes to your manuscript within the document by using the track changes mode in MS Word or by using bold or colored text.

Once the revised manuscript is prepared, you can upload it and submit it through your Author Centre.

When submitting your revised manuscript, you will be able to respond to the comments made by the reviewer(s) in the space provided.  You can use this space to document any changes you make to the original manuscript.  In order to expedite the processing of the revised manuscript, please be as specific as possible in your response to the reviewer(s).

IMPORTANT:  Your original files are available to you when you upload your revised manuscript.  Please delete any redundant files before completing the submission.

Because we are trying to facilitate timely publication of manuscripts submitted to the Oxford Open Neuroscience, your revised manuscript should be uploaded within the next 30 days from todays date.  If it is not possible for you to submit your revision in a reasonable amount of time, we may have to consider your paper as a new submission.

Once again, thank you for submitting your manuscript to the Oxford Open Neuroscience and I look forward to receiving your revision.

Sincerely,
Professor Sam Gilbert
Senior Editor, Oxford Open Neuroscience
sam.gilbert@ucl.ac.uk


Associate Editor
Comments to the Author:
(There are no comments.)

**Response to the Editor and Reviewer**

Dear Professor Gilbert,

Thank you very much for your careful reading of our revised manuscript.

We have addressed all points raised in your decision letter. Specifically, we removed all statements referring to the behavioural–neural correlation analysis from the Abstract and main text. We also corrected the wording regarding decoding accuracy by removing the term “above-chance” where applicable and updating the relevant text and figure legends accordingly.

All changes have been highlighted in the revised manuscript.

We appreciate your thoughtful comments and look forward to your decision.

Sincerely,
Michihiko Koeda

**Acceptance letter**

26-Feb-2026

Dear Dr. Koeda,

It is a pleasure to accept your manuscript entitled "Universal and Culture-Tuned Neural Codes for Vocal Emotion: An fMRI MVPA Study Using Japanese and Canadian Voices" in its current form for publication in Oxford Open Neuroscience.  

Please note that this journal operates with transparent peer review. This means that the full peer review history of your article will publish online alongside your article. This includes reviewer comments, editor decision letters, and your author responses.

Next steps
You will receive an email from no-reply@scipris.com within roughly one week. This is your invitation to sign up for an account with SciPris, Oxford University Press’ author portal hosted by Aptara. You will need to create an account if you do not already hold one. Please register or log into your account and follow the online instructions which will guide you through signing your licence and paying the APC. The email and the portal have clearly signposted support options if you need any help during this process.

Please note that SciPris is a completely different system from ScholarOne, so your credentials to submit your manuscript here will not work there. Once you’ve created a SciPris account, you will be able to use it whenever you publish with Oxford Open Neuroscience or any OUP journal. Please note that OUP will only ever request payment for applicable fees be made via SciPris or to an OUP bank account. If you ever have concerns about the legitimacy of a request, please do not hesitate to contact a customer services agent via the SciPris portal or directly via oupsupport@scipris.com.

Thank you for your fine contribution.  On behalf of the Editors of the Oxford Open Neuroscience, we look forward to your continued contributions to the Journal.

Sincerely,
Professor Sam Gilbert
Senior Editor, Oxford Open Neuroscience
sam.gilbert@ucl.ac.uk


Reviewer(s)' Comments to Author:
